# Evaluation of the *Candida* score as a mortality predictor in hospitalized patients with *Candida* spp. colonization: A retrospective cohort study

**DOI:** 10.5339/qmj.2025.84

**Published:** 2025-09-15

**Authors:** Luthvia Annisa, Mohamad S. Hakim, Abu T. Aman, Tri Wibawa

**Affiliations:** 1 Department of Microbiology, Faculty of Medicine, Public Health, and Nursing, Universitas Gadjah Mada, Yogyakarta, Indonesia; 2 Department of Biology and Immunology, College of Medicine, Qassim University, Buraydah, Saudi Arabia; 3 Laboratory of Clinical Microbiology, UGM Academic Hospital, Yogyakarta, Indonesia *Email: luthvia.annisa@ugm.ac.id

**Keywords:** *Candida*, colonization, infection, risk assessment, *Candidiasis*, invasive

## Abstract

**Background:**

Invasive candidiasis has risen alarmingly over the last decades, with diagnosis often complicated by colonization at multiple body sites. The *Candida* score offers a simplified tool to help identify patients at risk of invasive candidiasis, particularly in intensive care unit (ICU) settings; however, its applicability in patients with broader characteristics remains uncertain. Moreover, research on *Candida* colonization and the utility of such tools in Indonesia is still limited. This study aimed to evaluate the association between clinical and laboratory characteristics and patient outcomes, while also assessing the predictive value of the *Candida* score in both ICU and non-ICU patients at a tertiary academic hospital in Yogyakarta, Indonesia.

**Materials and Methods:**

This is an observational retrospective cohort study. During the period of January 2019 to December 2021, recorded laboratory data and clinical characteristics of patients (ICU and non-ICU patients) whose specimens yielded *Candida* spp. were collected through electronic health records. The correlation of laboratory profile and clinical characteristics with the outcome was analyzed comparatively using bivariate and multivariate analysis.

**Results:**

Ward status, length of stay, number of comorbid, history of steroids, *Candida* score of ≥3, and mechanical ventilation were significantly correlated with the non-survivor group. Multivariate analysis showed that intensive care status and a *Candida* score ≥3 were found to have the strongest and independent correlation with the non-survivor group (*p* ≤ 0.001; OR 11.8 [95% CI, 4.368–31.962] and *p* = 0.004; OR, 3.9 [95% CI, 1.562–9.065], respectively).

**Conclusion:**

Being in the ICU and having a *Candida* score of ≥3 was independently correlated with poorer clinical outcomes in hospitalized patients with *Candida* spp. colonization.

## 1. INTRODUCTION

*Candida* spp. are commonly found in the human body as part of the normal flora, starting from the oral cavity to the colon, skin, and female reproductive tract. Therefore, infections caused by *Candida* spp. are commonly derived from endogenous infections.^1^ The symptoms of *Candida* infections vary widely, ranging from superficial infection to systemic illness (invasive candidiasis).^2^ Overgrowth of *C. albicans* can result in opportunistic infections in numerous organs. Therefore, invasive illness is frequently caused by increased or aberrant colonization, as well as a localized or widespread absence of host defense.^1,2^

Invasive candidiasis is not a single clinical entity but rather a condition with a wide range of clinical symptoms that can affect any organ and remains a major concern, particularly among critically ill patients.^3^ Despite the development of several antifungal compounds within the last decades, overall crude mortality remains unchanged at ~40% in Europe.^3,4^

Invasive *Candida* infections are most commonly linked to candidemia (*Candida* species in the blood), affecting immunocompromised patients and those who require intensive care.^5^ Individuals with hematological malignancies (in patients who have just recovered from an episode of neutropenia), recipients of hematopoietic cell or solid organ transplants, and those given chemotherapeutic drugs for a range of disorders are all at risk for acquiring candidemia. Extensive gastrointestinal mucosal injury (such as after surgery), extensive burns, broad-spectrum antibiotics, and central venous catheters are also known risk factors of invasive candidiasis.^6,7^ In addition, due to the extensive development of interventional therapy for cancers, transplantations, and the overuse of antibiotics, the prevalence of invasive candidiasis has steadily increased.^8^ This rise highlights the need for improved awareness and timely intervention.

Early identification of patients who would develop invasive candidiasis remains a major problem in clinical settings. Several clinical prediction rules have been developed to identify high-risk patients with a high likelihood of developing invasive candidiasis, and who might best benefit from antifungal prophylaxis administration.^9,10^ Notably, a recent prospective study done by Poissy *et al* proposed a scoring system to assess risk factors of developing candidemia.^11^ While it poses a better predictive value, they rely on rigorous clinical and drug history, which might be too complicated to implement in a low-resource setting. *Candida* score, on the other hand, offers a more simplified approach and is widely recognized.^12^ However, the initial development and validation of the *Candida* score was based on patients hospitalized in the intensive care unit (ICU) and some of those requiring surgical intervention.^12,13^ Thus, its applicability in non-ICU and broader patient cohorts was questioned.^14^

In this study, we evaluated the use of *Candida* score in predicting clinical outcome in both ICU- and non-ICU-hospitalized patients, including those with and without surgical intervention. We further analyzed the correlation between the laboratory profile and the clinical characteristics with the outcome of patients (survivor vs. non-survivor) whose specimens yielded *Candida* spp. in a tertiary academic hospital in Yogyakarta, Indonesia.

## 2. MATERIALS AND METHODS

### 2.1 Study Design

This study was an observational, retrospective cohort study. The inclusion criteria for this study were: (1) inpatient status (both ICU and non-ICU patients), and (2) patients with a yeast culture positive for any *Candida* species followed by species-level identification. Patients were excluded if their medical records or laboratory data were incomplete, including—though not limited to—missing medical history.

A total sampling method was applied, enrolling all patients who met the inclusion and exclusion criteria within the study period. Using R Software^®^, we calculated a minimum required sample size of 79 subjects, based on an anticipated candidemia prevalence between 0.1% and 10%, with a 95% confidence interval and a 5% margin of error.

The study was conducted at a tertiary academic hospital in Sleman District, Special Region of Yogyakarta (Indonesia), using secondary data from the Laboratory of Clinical Microbiology and patients’ clinical characteristics from the electronic medical records from January 2019 to December 2021. Briefly, the data were collected from laboratory specimens and patient medical history using the electronic health record as part of SIMRS-UGM^®^ software. Data were documented in the form of tabulated Microsoft^®^ Excel files before being coded in the statistical software.

To maintain confidentiality, all enrolled patients were assigned a unique study ID. The study started only after ethical consideration was obtained. Ethical clearance was obtained from the institutional review board of the faculty and from the academic panel of the hospital, dated June 28, 2022, and July 21, 2022, respectively. The requirement for informed consent was waived due to the retrospective nature of the study.

### 2.2 Assessment of *Candida* Score

Each patient’s *Candida* score was recalculated during the data collection since the scoring was not routinely performed in our academic hospital. The *Candida* score is a clinical prediction rule used to predict the event of invasive candidiasis, proposed by Leon *et al*.^12^ This is a scoring system using the following parameters: severe sepsis or septic shock, multifocal colonization of *Candida*, total parenteral nutrition, and surgery (Table 1), originally with a cut-off of ≥2.5 and later revised to ≥3.^12^ However, since the term severe sepsis is no longer used, this study adopted the modification from Li *et al*,^5^ who used *Candida* score with a Sepsis 3.0 criteria of Sequential Organ Failure Assessment (SOFA) score of ≥2 as the definition of sepsis and found a comparable result.

### 2.3 Statistical Analysis

We used SPSS Statistics^®^ Software version 26 (IBM SPSS Inc., Chicago, IL) for the statistical analysis. Multivariate analyses using logistic regression were performed to evaluate the correlation between the patient’s clinical characteristics and the clinical outcome. Patients’ clinical characteristics were grouped based on known risk factors of invasive candidiasis, such as age, antibiotic exposure, and breach of mucosal integrity (e.g., through catheterization or mechanical ventilation). Differences in these characteristics were then analyzed between the survivor and non-survivor groups. Chi-square was used for categorical variables. The probability significance was expressed in *p*-value, statistical estimation was in confidence intervals (CI), and the degree of correlation was stated in odds ratio (OR). Candidates for multivariate analysis were derived from the bivariate analysis, in which the variables have a *p*-value of <0.25 and/or of substantive variables using a backward model. Additional analysis was conducted to evaluate the performance of the *Candida* score in predicting mortality using receiver operating characteristic (ROC) curve analysis. A one-way analysis of variance (ANOVA) and Tukey post-hoc test were used to determine the association between the length of hospital stay and *Candida* score.

## 3. RESULTS

During the study period, a total of 185 patients whose culture yielded *Candida* spp. were included for analysis. Based on the final outcome, they were grouped into survivors (*n* = 97, 52.4%) and non-survivors (*n* = 88, 47.6%). The majority of the subjects were from ICU (*n* = 125, 67.6%), of medical (non-surgical) diagnosis (*n* = 167, 90.3%), and the positive culture of *Candida* spp. were from non-sterile sites (*n* = 182, 98.4%), with sputum as the most common specimen type (*n* = 128, 69.2%) followed by urine (*n* = 51, 27.6%) then blood, pus, bronchoalveolar lavage, and pleural fluid (*n* = 2, 1.1%; *n* = 2, 1.1%; *n* = 1, 0.5%; and *n* = 1, 0.5%, respectively).

Of 185 isolates included, the three major *Candida* spp. isolated were *C*. *tropicalis*, *C*. *albicans*, and *C*. *parapsilosis* (*n* = 95 [51.4%]; *n* = 74 [40%]; and *n* = 4 [2.2%], respectively). For further analysis, the species of *Candida* were grouped into *C*. *albicans* (CA) and non-albicans *Candida* (NAC) groups.

### 3.1 The Correlation of Laboratory and Clinical Profile of Patients Whose Culture Yielded *Candida* spp. Towards the Outcome

Table 2 shows that statistically significant results were found from these variables with a positive correlation with the non-survivors group: ward status (*p* ≤ 0.001; OR, 11.556 [95% CI, 5.046–26.477]); length of stay (*p* = 0.049; OR, 2.88 [95% CI, 1.15–7.17]); number of comorbid (*p* = 0.033; OR, 3.052 [95% CI, 1.276–7.30]); history of steroid (*p* ≤ 0.001; OR, 2.959 [95% CI, 1.615–5.420]); *Candida* score (*p* ≤ 0.001; OR, 2.347 [95% CI, 1.766–3.012]); and mechanical ventilation (medical device status; *p* ≤ 0.001; OR, 3.381 [95% CI, 1.846–6.193]).

**Figure 1:**
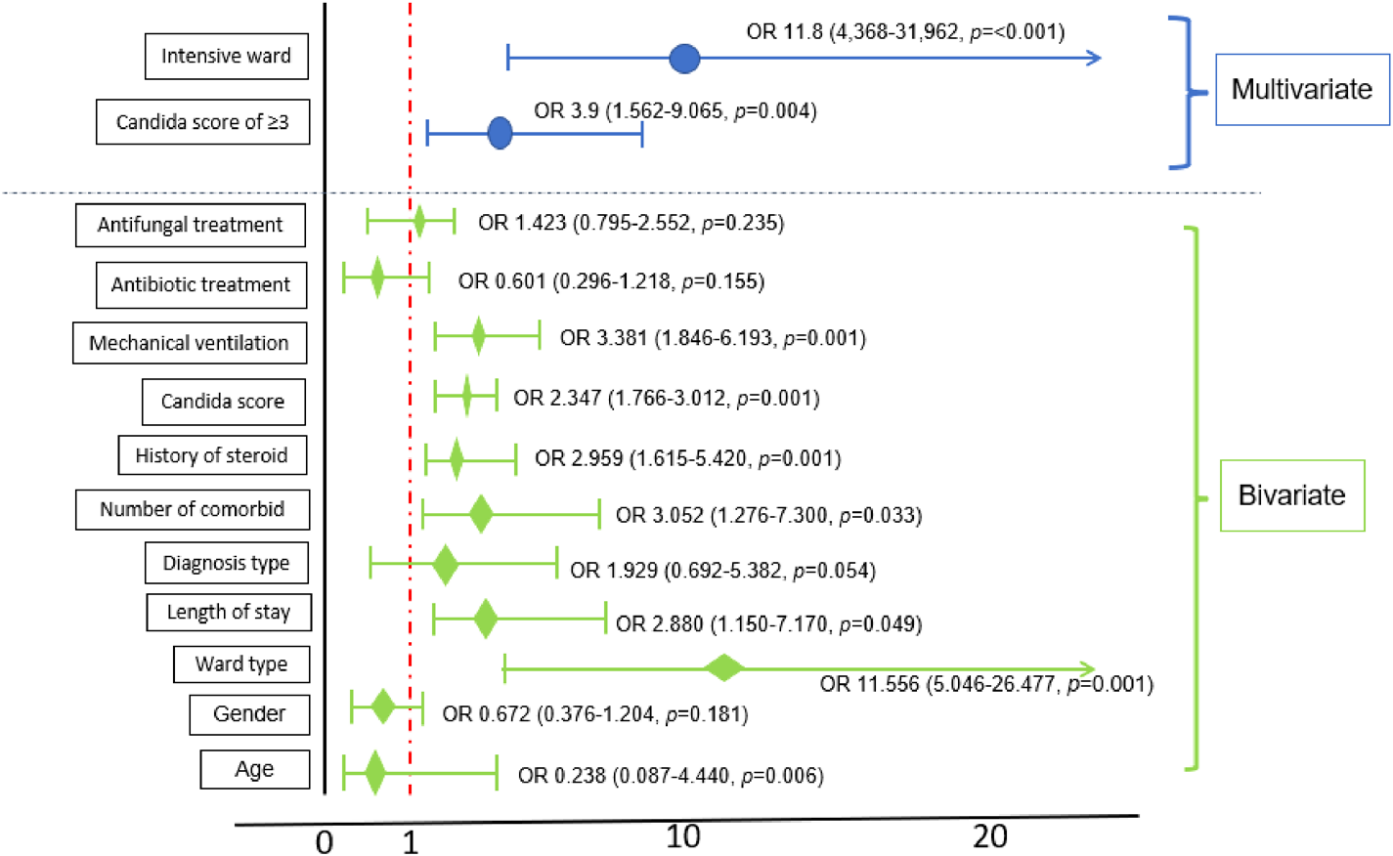
Multivariate logistic regression model of the patients’ profiles versus the outcome.

The variables that have the strongest correlation with the non-survivor outcome in a multivariate analysis were intensive care status and a *Candida* score of ≥3 (*p* ≤ 0.001; OR, 11.8 [95% CI, 4.368–31.962] and *p* = 0.004; OR, 3.9 [95% CI, 1.562–9.065], respectively; Figure 1).

Out of 185 patients enrolled, 24 patients (13%) were reported to have at least one bacterial coinfection. However, the number was not significantly different between the survivor and non-survivor groups (*p* = 0.798). The majority (*n* = 16/24, 67%) of bacterial pathogens cultured were Gram-negative bacteria (GNB), mainly *Klebsiella pneumoniae* and *Pseudomonas aeruginosa* (comprising 50% of 50% the identified GNB).

Among the total patients cultured positive for *Candida* spp., 103/185 patients (55.7%) received antifungal treatment during their hospital stay. Unfortunately, there was no significant difference in the outcome whether or not the patients received antifungal treatment (*p* = 0.235). We did further analysis, making antifungal treatment status the dependent variable and *Candida* score groups (scored <3 and ≥3) as the independent variable. Another statistically non-significant difference was found (*p* = 0.100), meaning in this study data set, the antifungal treatment given was independent of the *Candida* score status.

**Figure 2:**
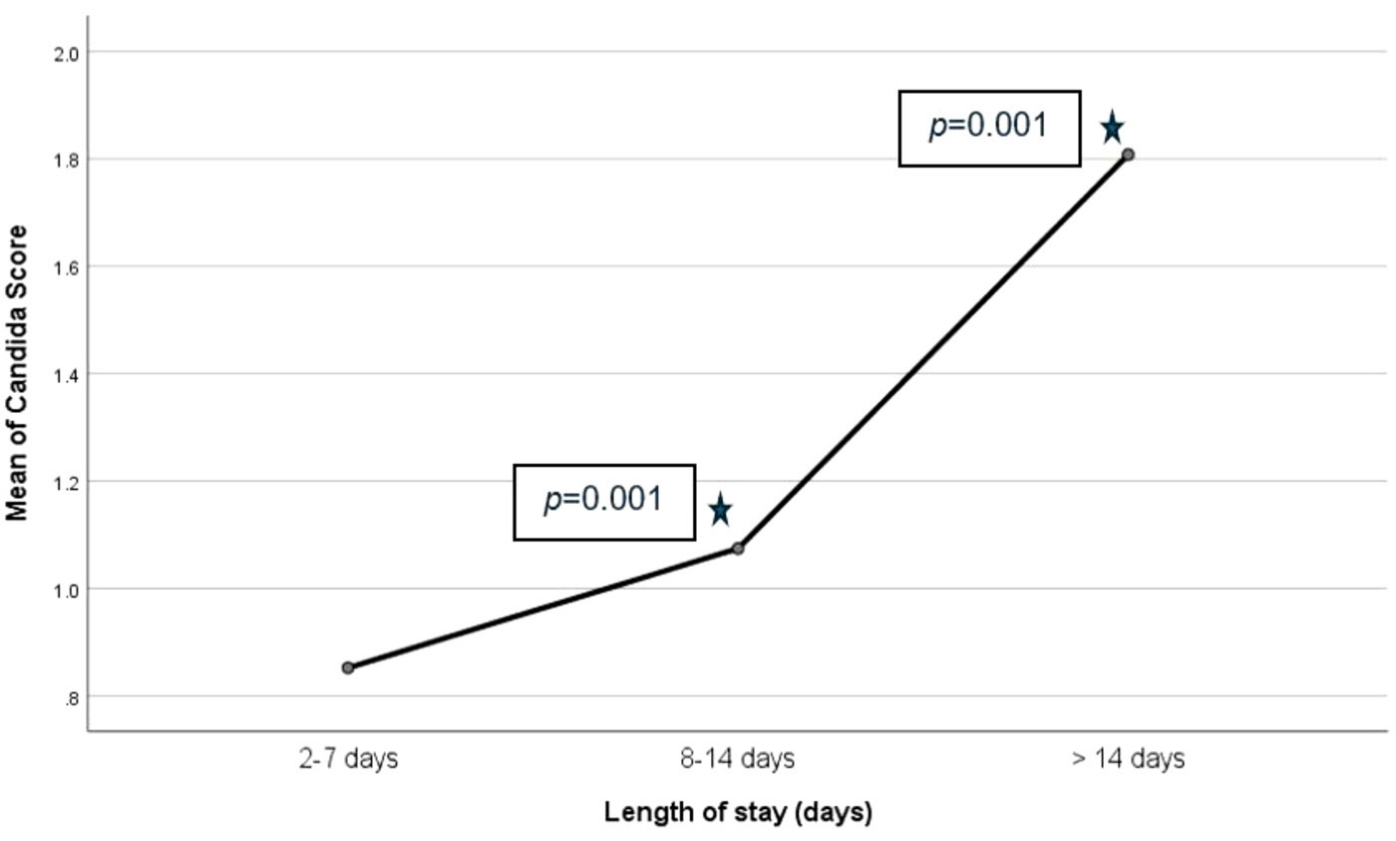
Association of hospital length of stay and Candida score. The longer the hospital stay, was significantly associated with the higher the Candida score in this study. Patients with a hospital stay of longer than 14 days had the highest mean Candida score compared to the other groups.

Patients’ hospital length of stay ranged from 4 to 73 days, and the mean hospital stay was 21.74 ± 17.05 days. For analysis, we grouped this into three groups: 2 to 7, 8 to 14, and >14 days groups, based on expected shifts in colonization risk, as prolonged hospitalization, particularly after the first week of admission, is known to increase the likelihood of skin and mucosal colonization with *Candida* species, as reviewed by Eggiman *et al*^15^ and Manzoni *et al*.^16^ The 8 to 14 days group showed statistically significantly higher *Candida* score compared to the 2 to 7 days group (*p* = 0.001), and the >14 days group had significantly higher *Candida* score compared with the 8 to 14 days group. The longer the hospital stay, the more significantly associated with the higher *Candida* score in this study (Figure 2).

### 3.2 The Performance of *Candida* Score

To evaluate the performance of the Candida score in this study, we did an ROC curve analysis to determine the area under the ROC curve (AUC), sensitivity, specificity, positive predictive value (PPV), and negative predictive value (NPV) of each *Candida* score in predicting mortality. An OR of each point toward the non-survivor outcome was also calculated using a logit prediction model. The detailed correlation was presented in Table 3.

**Figure 3:**
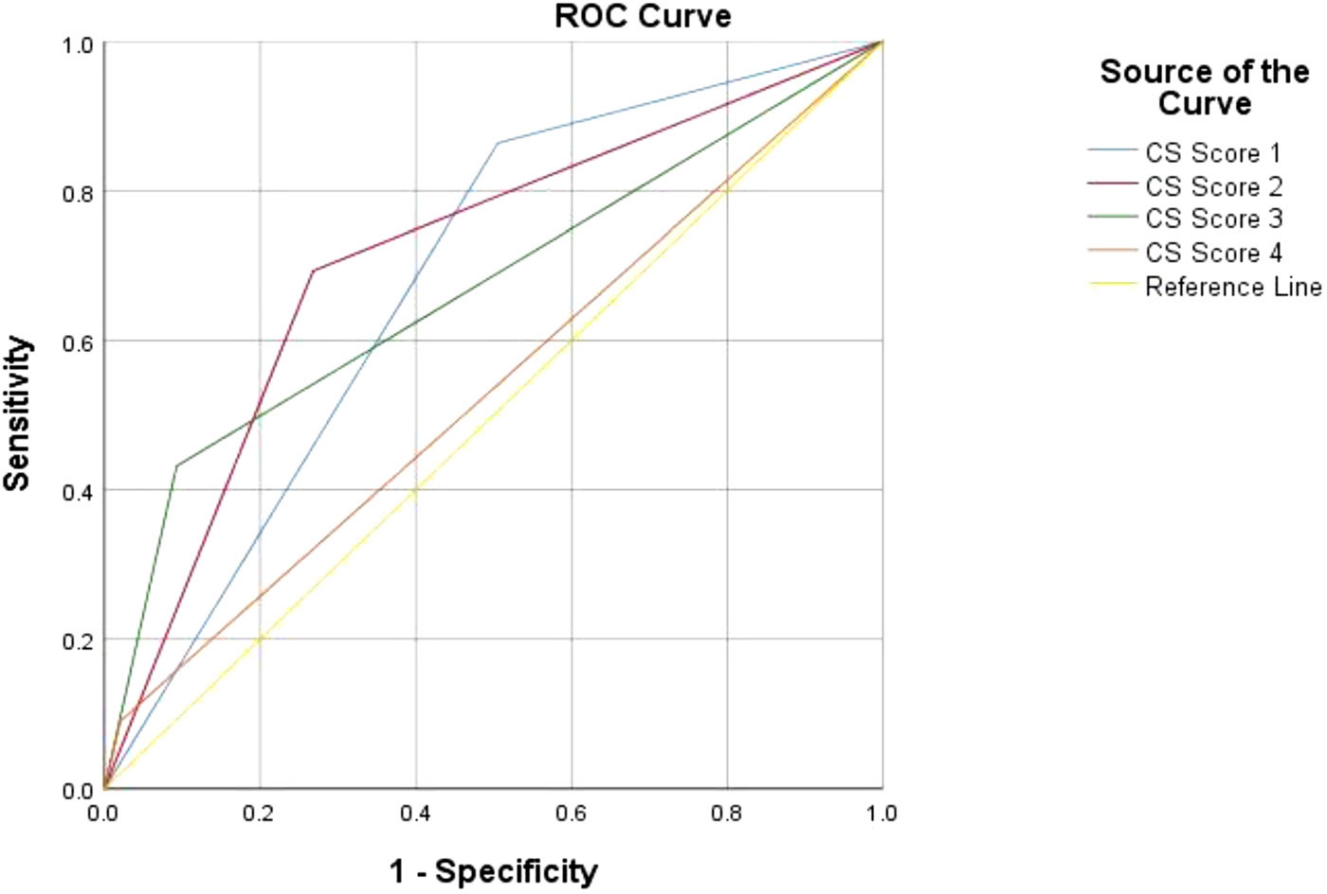
Receiver operating characteristic (ROC) curve of the Candida score as a mortality predictor. The highest area under the ROC curve (AUC) was shown by Candida score 2 (0.731); other parameters of Candida score performance in predicting mortality can be seen in Table 3.

Based on the ROC analysis of *Candida* score performance in predicting mortality, a mixed result was found. The highest AUC coverage belonged to *Candida* score 2 (0.713; Figure 3), followed by *Candida* score 1, 3, then 4 (0.679, 0.670, and 0.535, respectively). *Candida* score 1 showed the highest sensitivity (86.4%) and NPV (80.0%). While *Candida* score 4 was the most specific (specificity was 97.9%), the highest PPV was shown by *Candida* score 3 (80.8%). In addition, the higher the *Candida* score, the more likely one is to have a non-survivor outcome, as shown by the escalating OR for each score (Table 3).

This evaluation can be interpreted as follows: the lower score indicates a higher sensitivity but lower specificity, and vice versa. In order to increase a clinician’s awareness, a *Candida* score cut-off of 2 can be used as a screening point. However, a *Candida* score of 3 with its higher specificity, PPV, and OR that was not significantly different from score 4, was a reliable cut-off value to predict the poorer clinical outcome.

### 3.3 The Evaluation of Specimen Type as a Possible Confounding Variable

The diagnosis of candidiasis requires careful interpretation of the microbiological result in relation to the patient’s clinical status. In this study, the majority of specimens were from sputum, which commonly contained *Candida* spp. Hence, it is important to address the use of the *Candida* score in various specimen types in its correlation with the outcome. Thus, analysis of potential confounding variables was run toward the specimen type, specifically categorized as sputum versus non-sputum for this purpose. The analysis involved calculating the OR value when the specimen type was included versus when the specimen type was excluded. A difference of more than 10% is considered positive as a confounder. In this study, the delta is 1%; hence, the sputum specimen type was not a confounder to the *Candida* score performance in predicting the outcome.

ICU admission and a *Candida* Score of ≥3 were found as the strongest independent predictors of mortality in our data set. While other factors, such as the number of comorbidities, mechanical ventilation, and steroid use, were also associated with increased mortality, their significance diminished after adjustment for confounders. Interestingly, the *Candida* Score that was developed as a diagnostic tool for invasive candidiasis, also demonstrated its potential as a prognostic indicator for mortality, particularly at ≥3 cut-off. However, its broader application remains setting-specific, as reduced accuracy in non-ICU populations was also observed in other studies.^11,17^

## 4. DISCUSSION

Our study showed that being in the ICU and having a *Candida* score of ≥3 was independently correlated with poorer outcomes. However, clinical interpretations should be carefully followed through since the isolation of *Candida* spp. doesn’t necessarily conclude an infection, especially from naturally non-sterile specimens such as secretions of the respiratory tract, from which most specimens in this study were collected. The culture positive-*Candida* spp. identified in our study did not differentiate between colonization and infection. Therefore, the eventual event of “invasive candidiasis” was represented with the poorer clinical outcome.

A sputum specimen, whether expectorated or suctioned from the endotracheal tube, often heavily resided with *Candida* spp., and the frequency is increasing in critically ill patients (15% vs. 25%).^18^ Many studies have reported the importance of *Candida* colonization from any site in critically ill patients. *Candida* colonization is linked as an independent risk marker for developing invasive candidiasis in many studies. Lau *et al* reported that 84% of subsequent invasive candidiasis cases were successfully captured by screening the throat and perineal swab culture for *Candida* spp. using the *Candida* colonization index approach, a semiquantitative method for assessing patients’ *Candida* spp. colonization status.^19^

Even though the status of *Candida* spp. colonization and the need for antifungal intervention may still be far from an established guideline, but many studies have linked the *Candida* spp. colonization of the respiratory tract to worse outcomes in both immunocompetent and immunocompromised patients.^20^ Indeed, the immunocompetent patient groups were present with medical conditions that may impair their immune responses, such as the use of mechanical ventilation, a long hospital stay, and pre-existing cystic fibrosis.^21–23^ A systematic review by Zhang *et al* found that the development of invasive candidiasis was independently associated with the longer duration of ICU stay.^5^ In a study in India, an increased risk of developing *Candida* was associated with a longer hospital stay in both post-surgery and non-surgery cases.^24^ This supports the growing evidence that *Candida* spp. should be cautioned not only in hematological malignancy patients, but also in broader and more heterogeneous groups with the aforementioned medical conditions.

*Candida* spp. colonization was independently reported as a risk factor for the occurrence of acute Graft versus Host Disease (GVHD), irrespective of the patients’ Dectin-1 status.^25^ While Dectin-1 deficiency might result in diminishing patients’ immunity, leading to GVHD, the effect was observed to be indirect in addition to *Candida* spp. colonization status. This finding highlighted the role of fungal dysbiosis in the development of GVHD, the leading cause of mortality in post-transplant patients.^25^

In addition to being a co-factor in disease progression or a surrogate marker of poor patient regulation, another hypothesis is that *Candida* spp. colonization might eventually result in invasive systemic infections. The pathogenesis is better understood in *C*. *albicans*. Notorious for its morphologic and phenotypic plasticity, *C*. *albicans* hyphal switch and biofilm formation abilities are known to play an important role in inducing a systemic infection or complicating the co-infection with GNB.^26^ However, the host environmental factors, including pH, temperature, and nutrient availability, are also crucial in determining the expression of *C*. *albicans* virulence factors. Thus, the transition from commensalism to pathogenicity is not solely on the hyphal morphogenesis, but rather a complex cascade involving both the host and pathogen factors.^26^ It is also important to note that distinct levels of gene expression may exist in commensalism and pathogenicity of *Candida* spp. This different level then influences the growth of *Candida* spp. in pathogenic conditions in the colonized host.^27^

Another interesting theory of how *Candida* colonization is independently correlated with increased mortality is its known co-interaction with other pathogens, mainly GNB and *Staphylococcus* aureus. Many in vitro and animal studies reported that the co-existence of *Candida* spp. and *Pseudomonas aeruginosa* or *S*. *aureus* created a favorable niche for their survival and potentiated their ability to infect the host by delaying clearance of bacteria, forming polymicrobial biofilm, promoting each other’s virulence factors with the help of quorum sensing, and helping with immune evasion. However, further clinical evidence is needed in order to translate these results into antifungal therapy decision-making.^28–30^

Leon *et al*^12^ investigated the use of *Candida* score in a prospective study using adult, non-neutropenic, critically-ill patients population and reported that a *Candida* score ≥3 showed a more superior discriminatory power index compared with *Candida* Colonization Index with a cut-off of ≥0.5 in all aspects: area under ROC curve, sensitivity, specificity, NPV, PPV, and relative risk for developing invasive candidiasis, all were statistically significant results. The results for sensitivity and specificity numbers were considered too low to be used as a diagnostic tool, but a prominent result was shown for NPV (97.7%). Hence, the use of the *Candida* score is intended to exclude the possibility of a *Candida*-colonized, non-neutropenic, critically ill patient developing invasive candidiasis when the score is <3.

This study, however, showed a different outcome, with a *Candida* score cut-off of 3 having a superior performance in specificity and PPV. We hypothesized that it was because we compared it with mortality rather than as a diagnostic test for invasive candidiasis. Moreover, the logistic regression model showed that a *Candida* score of ≥3 had a 15-fold higher mortality risk.

Careful interpretation of *Candida* spp., a positive culture in the urine specimen, should also be conducted. Even though colonization always precedes a candidemia, a relatively low number of candidemia (5%–30%), versus 80% of *Candida* spp. colonization rate in critically ill patients showed its marked challenge when establishing a diagnosis of infection.^31^ However, the patient’s risk factors and clinical history should always be taken into consideration. Candiduria, whether symptomatic or asymptomatic, can be the only sign of disseminated candidiasis in approximately 80% of patients whose primary source of infection is from upper urinary tract. Some studies suggested requiring a second urine culture to rule out contamination.^32^ Again, applying laboratory results with clinical status is always of great importance. In high-risk patients developing fever or symptoms with unknown other sources while proven to have candiduria, then consideration for empirical antifungal therapy is not irrational.^33^

The Infectious Disease Society of America recommended echinocandin as the first-line therapy for both non-neutropenic and neutropenic clinically stable patients, along with source control management.^34^ Mortality was highly associated with the timing of the antifungal given and appropriate source control.^35^ Candidemia was often linked with a central venous catheter in non-neutropenic patients. However, gastric tubes were often predominated as a source of disseminated candidiasis in neutropenic patients.^34^ In intra-abdominal candidiasis, an appropriate source control through debridement and drainage within 5 days of positive *Candida* culture along with antifungal treatment was correlated with a significantly reduced mortality rate.^36^

This study found a contradictory result regarding antifungal treatment given and the patients’ outcome, as it showed no significant differences in mortality whether or not the patient received antifungal treatment during their hospital stay. We suggest this could be caused by the diagnosis of candidiasis was not established, in addition to the lack of source control data to draw a correlation analysis. However, even though a diagnosis of invasive candidiasis was not established in this study, many studies have shown the importance of *Candida* colonization in high-risk patients. Invasive candidiasis was highly correlated with an increasing amount of *Candida* spp. colonization, proven through semi-quantitative cultures, as opposed to non-colonized patients.^37^ Our study also showed that the decision to give antifungal treatment to patients was independent of the *Candida* score, while the *Candida* score of ≥3 itself was shown to be independently correlated with mortality among critically ill patients in our multivariate analysis. This showed that in a limited resources setting, where diagnostic tests are still scarce, establishing invasive candidiasis is even more challenging and may lead to underdiagnosed cases. This finding highlights the need to implement the use of an applicable algorithm or scoring system in a hospital to direct clinical decisions in suspected *Candida* infections.

In summary, this study found that the same sentiment regarding the use of *Candida* colonization status, a microbiological parameter, combined with an understanding of other clinical profiles of patients, served as a good predictor of the subsequent event that led to mortality. Our results should encourage further, large-enrollment, multi-site, prospective clinical studies looking at the correlation between the clinical characteristics and the status of *Candida* spp. colonization in high-risk patients.

## 5. LIMITATIONS

There are several limitations in this study. First, it was performed in a retrospective manner, where selection bias may occur. Second, the sample size was relatively small, and certain populations, such as post-surgery and neutropenic patients, could not be considered representative due to the small percentage. Lastly, due to the limited information on patients’ clinical history and the non-definite status of colonization or infection, reevaluation of the invasive candidiasis diagnosis could not be established. Hence, the correlation between antifungal treatment administered and/or source control measures could not be assessed. As reflected in the discussion above, this may affect the clinical judgement in interpreting the results of this study.

## 6. CONCLUSION 

A significant correlation between clinical characteristics and outcomes was observed. In this study, patients whose culture yielded *Candida* spp. and had any of these characteristics: intensive care status, length of stay ≥14 days, having two or more comorbidities, a history of steroid ≥14 days, a *Candida* score of ≥3, and on mechanical ventilation were more likely to have a poorer outcome. Our study showed that being in the ICU and having a *Candida* score of ≥3 was independently correlated with poorer outcomes. However, clinical interpretations should be carefully followed through since the isolation of *Candida* spp. doesn’t necessarily conclude an infection, especially from naturally non-sterile specimens. A multivariate analysis found that intensive care status and a *Candida* score of ≥3 are independently associated with poorer outcomes. Thus, a simple scoring system such as the *Candida* score, which combines the *Candida* spp. colonization status with other patients’ clinical profiles is beneficial in directing further clinical decisions. A positive *Candida* colonization in an ICU setting, particularly among mechanically ventilated patients, is information that should not be overlooked, but rather interpreted cautiously.

## Figures and Tables

**Table 1.** *Candida* score scoring system, adapted from Leon et al.^2^

Parameters of *Candida* score	Score if present	Score if absent
Clinical sepsis: severe sepsis or septic shock	2	0
Total parenteral nutrition	2	0
Multifocal colonization	1	0
Surgery	1	0

**Table 2.** Bivariate analysis of the patient’s laboratory and clinical profile versus the outcome.

No.	Laboratory and clinical profiles		Outcome						Total	OR	CI 95% (upper–lower bound)		*p* value	
			Non-survivor (*n* = 88)	%	Survivor (*n* = 97)	%								
1	Candida grouped	CA	56	50.5	55	49.5			111	1.336	0.740–2.415		0.336	
		NAC	32	43.2	42	56.8			74					
2	Age	1–17 years old	3	25	9	75			12	0.238	0.087–4.440		0.006	
		18–60 years old	35	64.8	19	35.2			54					
		>60 years old	50	42	69	58			119					
3	Gender	Male	44	43.1	58	56.9			102	0.672	0.376–1.204		0.181	
		Female	44	53	39	47			83					
4	Ward	Intensive	80	64	45	36			125	11.556	5.046–26.477		<0.001	
		Non-intensive	8	13.3	52	86.7			60					
5	Length of stay	2–7 days	8	29.6	19	70.4			27	2.880	1.15–7.170		0.049	
		8–14 days	23	42.6	31	57.4			54					
		>14 days	57	54.8	47	45.2			104					
6	Specimen type	Sterile	1	33.3	2	66.7			3	0.546	0.049–6.128		0.619	
		Non-sterile	87	47.8	95	52.2			182					
7	Diagnosis type	Medical	82	49.1	85	50.9			167	1.929	0.692–5.382		0.306	
		Surgical	6	33.3	12	66.7			18					
8	Number of comorbid	No	28	37.3	47	62.7			75	3.052	1.276–7.300		0.033	
		One comorbid	40	50.6	39	49.4			79					
		Two or more	20	64.5	11	35.5			31					
9	Neutropenia	Yes	1	33.3	2	66.7			3	0.546	0.049–6.128		1.000	
		No	87	47.8	95	52.2			182					
10	History of steroids	Yes	61	59.2	42	40.8			103	2.959	1.615–5.420		<0.001	
		No	27	32.9	55	67.1			82					
11	Candida Score at specimen collection	Score of ≥3	38	80.9	9	19.1			47	2.347	1.766–3.012		<0.001	
		Score of <3	50	36.2	88	63.8			138					
12	Bacterial coinfection	Yes	12	50.0	12	50.0			24	1.118	0.474–2.637		0.798	
		No	76	47.2	85	52.8			161					
13	Medical devices													
	Central venous catheter	Yes	10	55.6	8	44.4	18	1.426		0.536–3.793		0.641
		No	78	46.7	89	53.3	167					
	Mechanical ventilation	Yes	54	63.5	31	36.5	85	3.381		1.846–6.193		<0.001
		No	34	34	66	66	100					
14	Total days of antibiotic use	≥10 days	23	57.5	17	42.5	40	0.601		0.296–1.218		0.155
		<10	65	44.8	80	55.2	145					
15	Antifungal treatment given	Yes	53	51.4	50	48.6	103	1.423		0.794–2.552		0.235
		No	35	42.7	47	57.3	82					

**Table 3.** The evaluation of *Candida* score performance in predicting mortality in the non-survivor group.

	Area under the receiver operating characteristic curve	Sensitivity (%)	Specificity (%)	Positive predictive value (%)	Negative predictive value (%)	Predictive analysis		
						Odds ratio	95% CI (lower–upper bound)	*p* value
*Candida* score of 1	0.679	86.4	49.5	60.8	80.0	2.608	1.052–6.436	0.038
*Candida* score of 2	0.713	69.3	73.2	70.1	72.4	5.411	2.221–13.186	<0.001
*Candida* score of 3	0.670	43.2	90.7	80.8	63.8	15.000	5.495–40.945	<0.001
*Candida* score of 4	0.535	9.1	97.9	80.0	54.3	16.000	3.001–85.304	0.001
